# Human Adipose-Derived Mesenchymal Progenitor Cells Engraft into Rabbit Articular Cartilage

**DOI:** 10.3390/ijms160612076

**Published:** 2015-05-27

**Authors:** Wen Wang, Na He, Chenchen Feng, Victor Liu, Luyi Zhang, Fei Wang, Jiaping He, Tengfang Zhu, Shuyang Wang, Weiwei Qiao, Suke Li, Guangdong Zhou, Li Zhang, Chengxiang Dai, Wei Cao

**Affiliations:** 1 Cellular Biomedicine Group, Palo Alto, CA 94301, USA; E-Mails: maxwell.wang@cellbiomedgroup.com (W.W.); elena.he@cellbiomedgroup.com (N.H.); iris.feng@cellbiomedgroup.com (C.F.); victor.liu@cellbiomedgroup.com (V.L.); lizzy.zhang@cellbiomedgroup.com (L.Z.); richard.wang@cellbiomedgroup.com (F.W.); jeffrey.he@cellbiomedgroup.com (J.H.); maggie.li@cellbiomedgroup.com (S.L.); helen.zhang@cellbiomedgroup.comchase.dai@cellbiomedgroup.com (C.D.); 2 Department of Pathology, Shanghai Medical College, Fudan University, Shanghai 200433, China; E-Mails: zhutengfang@shmu.edu.cn (T.Z.); wangshuyang@shmu.edu.cn (S.W.); 3 Animal facility, Shanghai Medical College, Fudan University, Shanghai 200433, China; E-Mail: qiaoweiwei@shmu.edu.cn; 4 Department of Plastic and Reconstructive Surgery, Shanghai 9th People’s Hospital, Shanghai Jiao Tong University School of Medicine, Shanghai 200011, China; E-Mail: guangdongzhou@126.com; 5 Department of Histology and Embryology, Shanghai Medical College, Fudan University, Shanghai 200433, China

**Keywords:** mesenchymal stem cells, human adipose-derived mesenchymal progenitor cells, osteoarthritis, human leukocyte antigen

## Abstract

Mesenchymal stem cells (MSCs) are known to have the potential for articular cartilage regeneration, and are suggested for the treatment of osteoarthritis (OA). Here, we investigated whether intra-articular injection of xenogeneic human adipose-derived mesenchymal progenitor cells (haMPCs) promoted articular cartilage repair in rabbit OA model and engrafted into rabbit articular cartilage. The haMPCs were cultured *in vitro*, and phenotypes and differentiation characteristics of cells were evaluated. OA was induced surgically by anterior cruciate ligament transection (ACLT) and medical meniscectomy of knee joints. At six weeks following surgery, hyaluronic acid (HA) or haMPCs was injected into the knee joints, the contralateral knee served as normal control. All animals were sacrificed at the 16th week post-surgery. Assessments were carried out by macroscopic examination, hematoxylin/eosin (HE) and Safranin-O/Fast green stainings and immunohistochemistry. The data showed that haMPC treatment promoted cartilage repair. Signals of human mitochondrial can be directly detected in haMPC treated cartilage. The haMPCs expressed human leukocyte antigen I (HLA-I) but not HLA-II-DR *in vivo*. These results suggest that intra-articular injection of haMPCs promotes regeneration of articular cartilage in rabbit OA model, and support the notion that MPCs are transplantable between HLA-incompatible individuals.

## 1. Introduction

Osteoarthritis (OA) is a chronic age-related disease of the whole joint characterized by the slowly progressive destruction of articular cartilage accompanied by hyperplasia of synovium, osteosclerosis of subchondral bone, retrogression of cruciate ligaments, degeneration of menisci, hypertrophy of the joint capsule. According to the evidence-based guideline of American Academy of Orthopaedic Surgeons (AAOS), treatment for OA including glucosamine, chondroitin, acupuncture, physical agents, and lateral wedge insoles were not recommended while the only pharmacologic treatment recommended for OA was nonsteroidal anti-inflammatory drugs (NSAIDs; oral or topical) or tramadol for patients with symptomatic OA of the knee, merely providing symptomatic relief from pain and failing to prevent cartilage damage and subsequent destruction of other joint tissues [1]. In recent years, New England Journal of Medicine published a series of results of controlled clinical trials demonstrating little effect of arthroscopic surgery for the treatment of OA [2,3,4].

Cell therapy by surgical autologous chondrocyte implantation (ACI) has been used to regenerate cartilage defects for more than 20 years [5]. However, when comparing ACI with other classical treatment, especially microfracture, in 80 patients, no significant differences were found between the methods after 24 months’ follow-up [6]. Furthermore, when evaluated the efficacy of microfracture and characterized chondrocyte implantation (CCI) in 118 patients, the overall Knee injury and Osteoarthritis Outcome Score (KOOS) was not different between the interventions at the five year follow-up, although at the 36 month follow-up, CCI showed significant improvement [7].

New strategy, mesenchymal progenitor cells (MPCs) are known to have a potential for articular cartilage regeneration [8,9,10]. MPCs can be obtained from different sources including bone marrow, adipose tissue, dental pulp, umbilical cord blood, synovial membrane, placenta, skin, umbilical cord perivascular cells, skeletal muscle, Wharton’s jelly, meniscus, breast milk, cartilage, ligament, and fat pad. Compared with bone marrow-derived MPCs (BM-MPCs), adipose tissue-derived MPCs (AD-MPCs) are more genetically stable in a long term culture, display a lower senescence ratio and higher proliferative capacity [11]. Recently, some studies evaluating autologous AD-MPC therapy showed that AD-MPCs improved function and pain of the knee joint without causing adverse events, and reduced cartilage defects by regeneration in OA pre-clinically or clinically [10,12,13].

MPCs express human leukocyte antigen (HLA) major histocompatibility complex (MHC) class I molecule and negligibly low HLA-II molecules [14,15]. After intrauterine injection of xenogeneic human BM-MPCs into sheep, the cells engraft and differentiate into multiple mesenchymal lineages. Unexpectedly, long-term engraftment occurred after the fetuses became immunocompetent [16]. In addition, other experiments demonstrated that implantation of allogeneic, major histocompatibility-mismatched MPCs into baboons has been well tolerated and *in vitro* study also found that addition of MSCs in primary mixed lymphocyte cultures may suppress T-cell proliferation [17,18,19]. Furthermore, it has been shown that AD-MPCs possess immunosuppressive capability and can be xenogeneically transplanted in immunocompetent recipients without the use of immunosuppressants [20].

To date, transplantation of xenogeneic human adipose-derived MPCs (haMPCs) has not been fully investigated. In our present study, MPCs were separated from human adipose tissue, expanded *in vitro* and then injected into rabbit knee joints. The objective of this study was to determine whether intra-articular injection of haMPCs promoted the repair of cartilage in rabbit OA model and engrafted into rabbit articular cartilage. To the best of our knowledge, it is the first study to deliver xenogeneic haMPCs into the knee joints of rabbit osteoarthritis.

## 2. Results

### 2.1. Characterization of haMPCs

**Figure 1:**
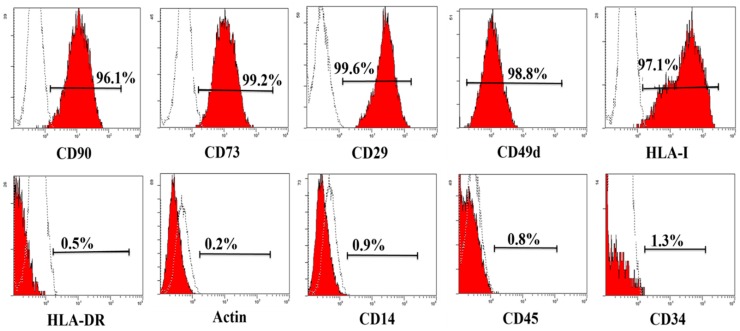
Phenotype of haMPCs. The haMPCs are positive for CD90, CD73, CD29, CD 49d and HLA-I, negative for CD45, CD14, CD34, HLA-DR and Actin.

**Figure 2:**
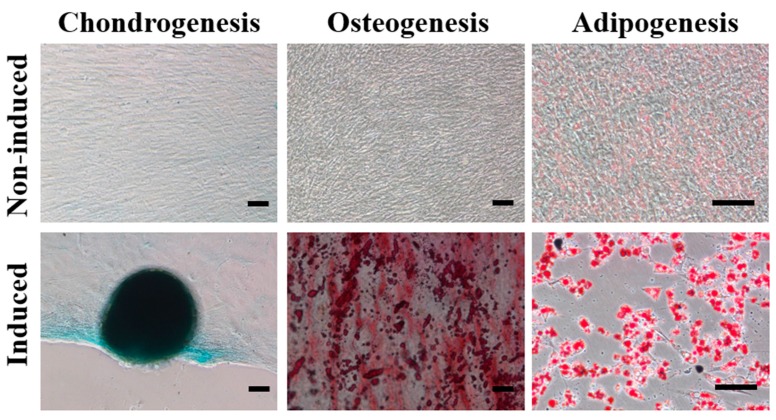
Differentiation of human adipose-derived mesenchymal progenitor cells (haMPCs). Multipotent differentiation of haMPCs showed positive staining toward chondrogenesis (Alcian Blue), osteogenesis (Alizarin Red) and adipogenesis (Oil Red O). Scale bars = 50 μm.

MPCs are defined retrospectively by characteristics *in vitro*, including a combination of phenotypic markers and multipotential differentiation functional properties [21]. To begin our study of the effects of haMPCs on the OA model, we first examined the characteristics of haMPCs. haMPCs were isolated, expanded and characterized by flow cytometry. The results showed that haMPCs were positive for CD90, CD73, CD29, CD49d and HLA-I, while cells were negative for HLA-DR, Actin, CD14, CD45, CD34 (Figure 1). In addition, chondrogenesis of expanded haMPCs was confirmed by Alcian Blue staining, osteogenesis as determined by Alizarin Red staining and adipogenesis as assayed by Oil Red O staining (Figure 2). These results demonstrate that the cells were confirmed to the characterization of AD-MPCs.

### 2.2. haMPC Treatment Promotes Articular Cartilage Repair

**Figure 3:**
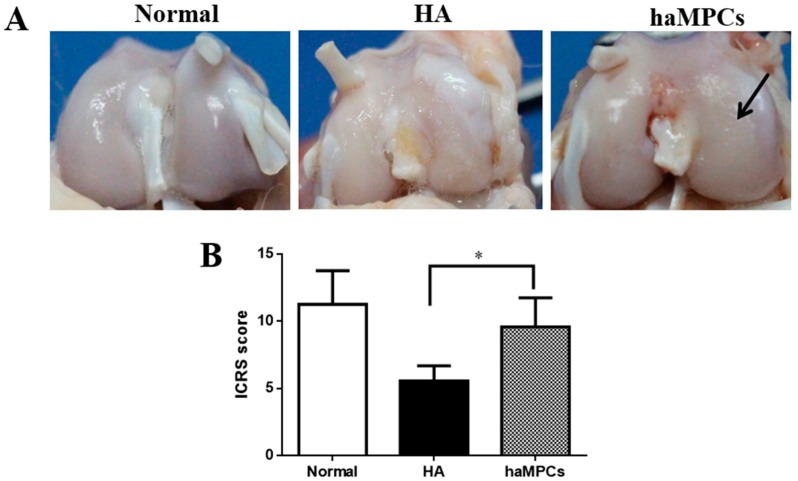
Macroscopic findings. (**A**) Sixteen weeks after surgery, in haMPC treatment group, eroded cartilage was almost completely covered by the repaired tissues (black arrow); and (**B**) Macroscopic OA ICRS scores showed haMPC treatment significantly increased ICRS scores compared with hyaluronic acid (HA) group. * indicates *p <* 0.05.

Macroscopically, 16 weeks after surgery, macroscopic evaluation exhibited that eroded cartilage of haMPC treatment was almost completely covered by the repaired tissues, and the repaired cartilage surface was relatively smooth. While large fissures and cracks were observed in hyaluronic acid (HA) treatment group as control compared with the cartilage of normal group (Figure 3A). The macroscopic OA ICRS scores were significantly higher in haMPC group than the HA group (Figure 3B).

**Figure 4:**
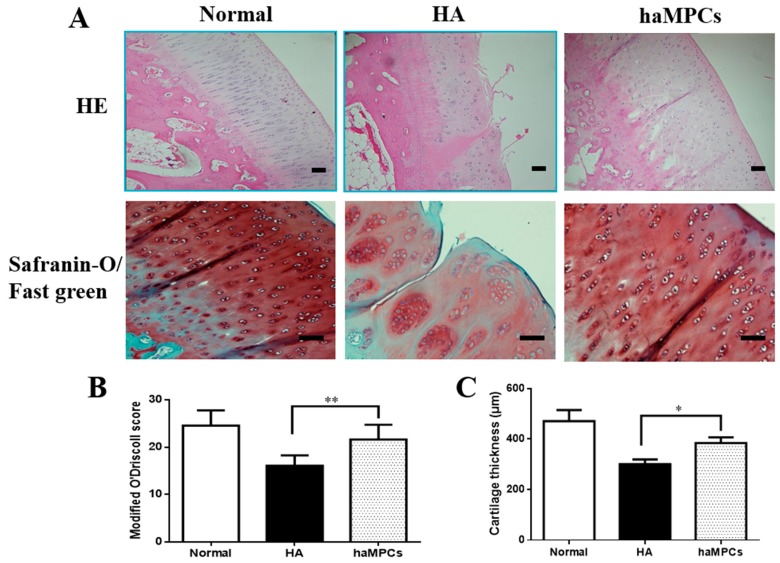
Histological findings. (**A**) HE and Safranin-O/Fast green stainings showed that haMPC treatment alleviated fissures and cracks formation; (**B**) The modified O’Driscoll histological score showed that haMPC treatment had significantly higher score compared with HA group; and (**C**) The haMPCs significantly increased cartilage thickness compared to HA group. Scale bars = 50 μm. * indicates *p <* 0.05; ** indicates *p <* 0.01.

Histologically, in normal group, HE staining demonstrated that the surface of normal articular cartilage was smooth and had no fissures and cracks. Superficial zone, mid zone, deep zone, and calcified cartilage were all distinct. The superficial zone of cartilage and the tidemark between the deep zone of and calcified cartilage were intact and clear. In HA group, the articular cartilage had a rough border showing fibrillation formation and the superficial zone of chondrocytes was fragmentary. However, haMPC treatment revealed few fissures, few cracks, and almost continuous superficial zone (Figure 4A).

Safranin-O/Fast green staining clearly revealed the progression of degenerative OA changes in HA group compared with normal group. In particular, superficial fibrillation, proteoglycan depletion, extension of crack, and articular cartilage reduction were observed. However, the size and quantity of clusters were reduced and proteoglycan expression increased in haMPC group. The treatment with haMPCs led to the formation of a new cartilage tissue: smooth surface, a higher presence of proteoglycan, a decreased Fast green staining in superficial zone of cartilage compared with HA group, indicating that decreased collagen I expression was shown in haMPC treatment (Figure 4A).

Furthermore, the modified O’Driscoll histological score revealed that haMPC treatment had significantly higher score compared to HA group (Figure 4B). Cartilage thickness increased significantly from 324 μm in HA group to 400 μm in haMPC group, respectively (Figure 4C). Taken together, these results suggested that haMPC treatment promoted cartilage regeneration.

### 2.3. haMPC Treatment Increased Collagen II Expression and Decreased MMP-13 Expression

**Figure 5:**
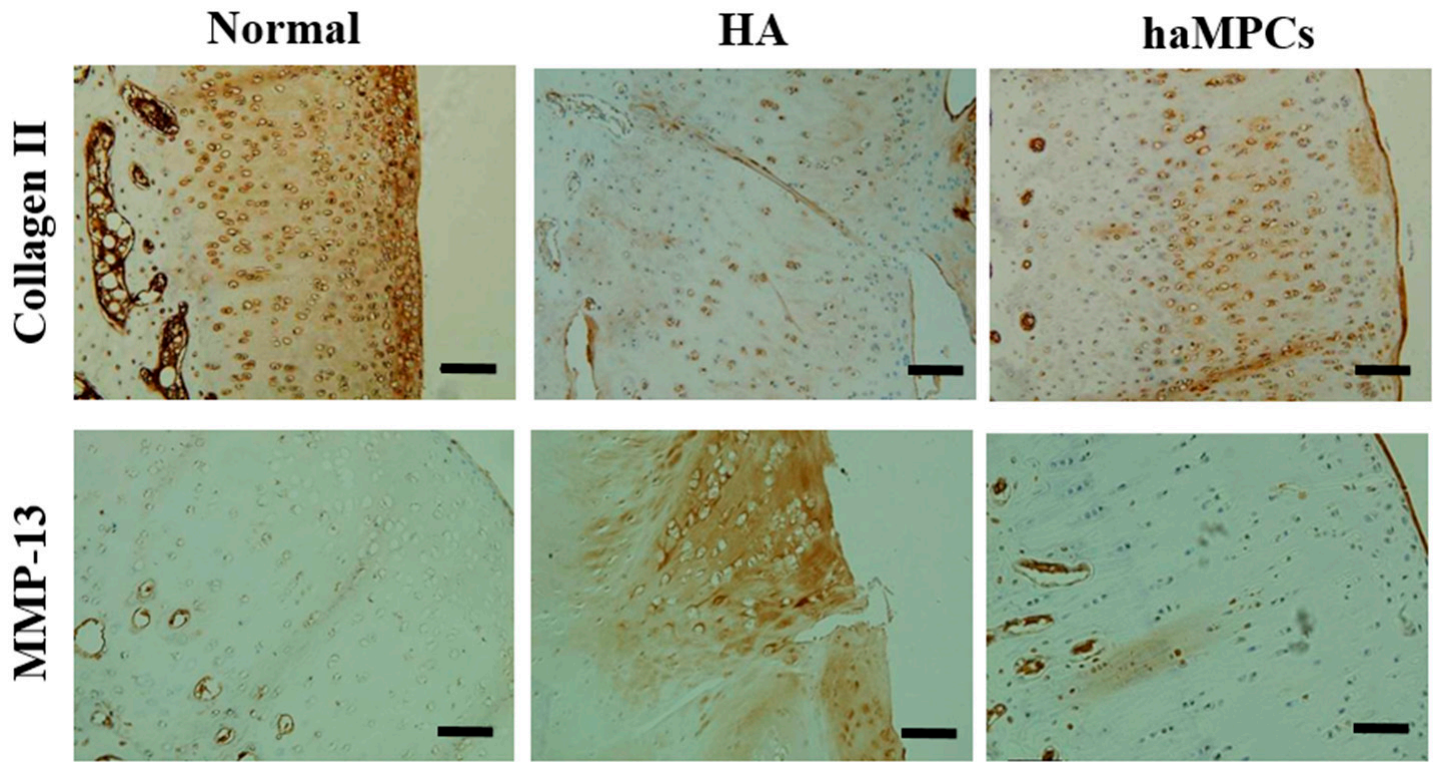
Immunostaining of type II collagen and MMP-13. The haMPC treatment increased articular cartilage type II collagen expression and decreased articular cartilage MMP-13 secretion. Scale bars = 50 μm.

We found haMPC treatment promoted cartilage repair, and the next step we further investigated whether the repaired cartilage expressed hyaline cartilage specific marker, type II collagen. Immunohistochemical detection displayed that in normal articular cartilage, collagen II distributed especially high at superficial zone of cartilage and expressed evenly at the mid and deep zones of cartilage. In HA group, collagen II expressed very weakly and less extensively around the chondrocyte-like cells compared to the normal cartilage. However, collagen II expression was stronger and more extensive around the chondrocyte-like cells in haMPC group compared with HA group (Figure 5).

Since global knockout *Mmp-13^−/−^* mice showed inhibition of cartilage erosion, this study indicated that matrix degradation during OA may be caused by aggrecanases [22]. Therefore, we also examined the expression of collagen II specific degrading enzyme, matrix metalloproteinase-13 (MMP-13). Results showed that MMP-13 was negative in normal cartilage. However, in HA group, MMP-13 displayed a diffuse and extensive dark-brown positivity around chondrocytes and extracellular matrix of superficial layer, especially near the area of fissures and cracks. By contrast, haMPC group showed obvious reduction in expression of MMP-13 (Figure 5). These results indicate that haMPC treatment inhibits cartilage degeneration.

### 2.4. The haMPCs Engrafted into Rabbit Cartilage

**Figure 6:**
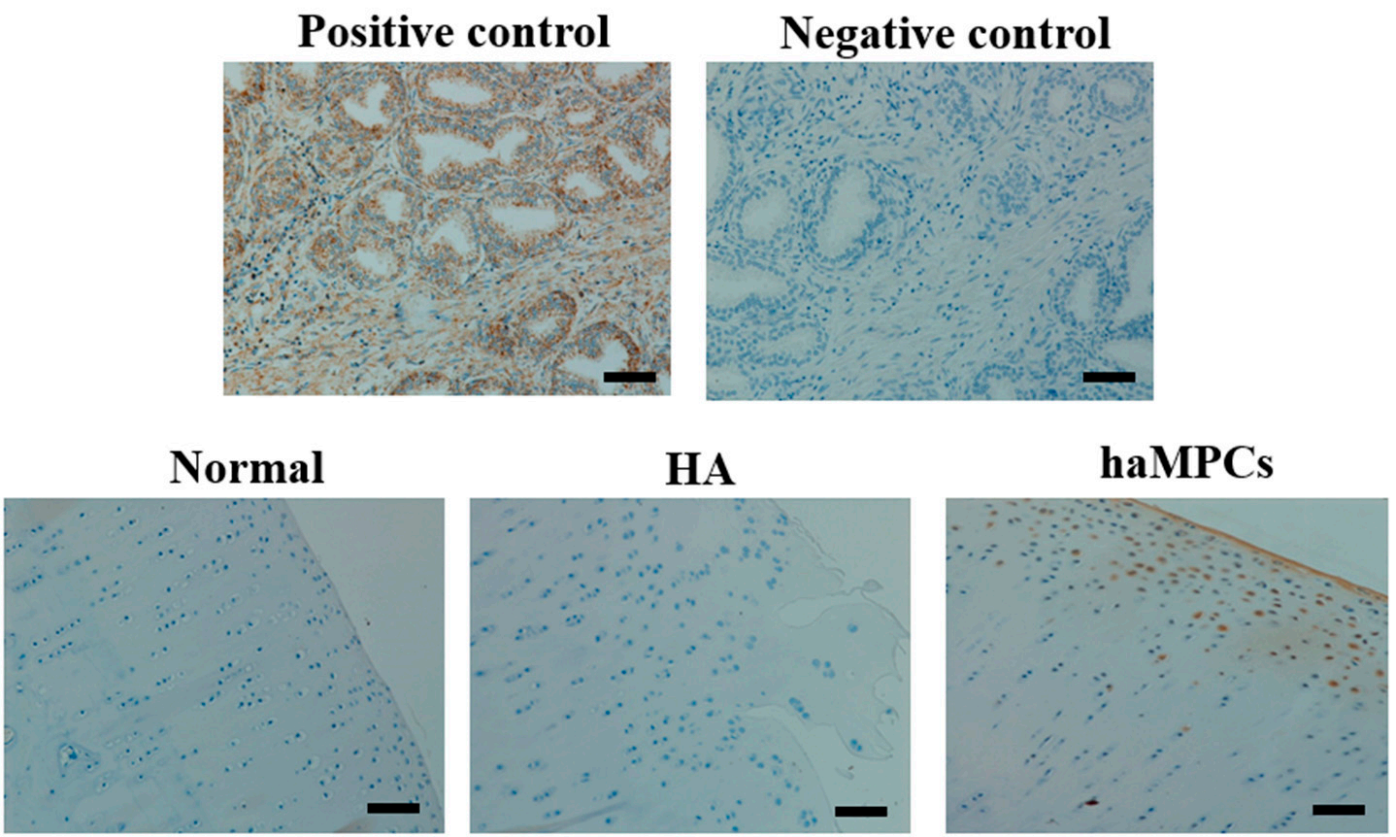
haMPCs engrafted into rabbit cartilage. In normal and HA treated cartilage, human mitochondrial signal was negative. In haMPC group, human cells were engrafted into rabbit cartilage. Immunohistochemical staining in human prostate tissue was used as positive control and negative (isotype) control. Scale bars = 50 μm.

The next step, we want to know whether injected haMPCs engraft into rabbit cartilage. Using anti-human monoclonal mitochondrial antibody, immunohistochemical staining indicates that human mitochondrial signal was positive in haMPC treatment group, whereas it was negative in normal and HA groups (Figure 6). These results indicate that haMPCs directly engraft into the regenerated cartilage.

### 2.5. HLA-I but Not HLA-II-DR Expressed by haMPCs in Vivo

**Figure 7:**
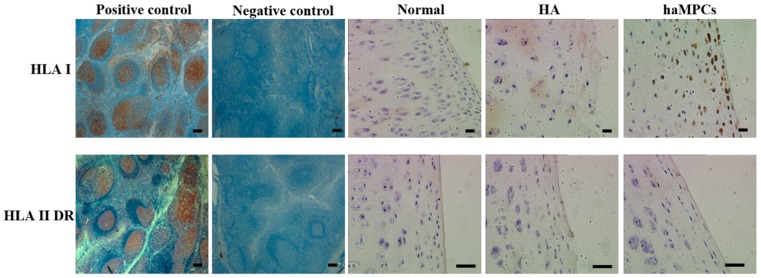
HLA-I but not HLA-II-DR was expressed in haMPC treatment group. In normal and HA treated cartilage, HLA-I and HLA-II-DR were negative. HLA-I but not HLA-II-DR was expressed in haMPC treated cartilage. Immunohistochemical analyses of HLA-I and HLA-II-DR in human tonsil tissue were used as positive control and negative (isotype) control. Scale bars = 50 μm.

Since we found haMPCs engraft into rabbit cartilage, the next step we want to know whether engrafted haMPCs express HLA-I and HLA-II-DR *in vivo*. Using anti-human monoclonal antibodies, we found engrafted haMPCs express HLA-I but not HLA-II-DR (Figure 7), which are consistent with *in vitro* phenotypic data (Figure 1). Moreover, HLA-I positive cells locate at the superficial area of rabbit cartilage consistent with engraftment data (Figure 7).

## 3. Discussion

Traditionally, bone marrow is regarded as richest and most reliable reservoir for MPCs; however, only 0.001%–0.01% of mononuclear cells in bone marrow are MPCs while MPCs constitute up to ~2% within the stromal vascular fraction [23]. Moreover, harvesting MPCs from bone marrow is a highly invasive procedure causing pain, morbidity of the donor sites and further rehabilitative burden on the patient compared with harvesting MPCs from lipoaspirates. Furthermore, differentiation potential of MPCs from bone marrow declines with aging whereas AD-MPCs have shown comparable differentiation capability during aging [24]. In the meantime, the number of adipose-derived MPC-based product Investigational New Drugs (INDs) submitted to FDA and clinical trials registered worldwide increased significantly from 2011 indicating that adipose tissue is becoming an attractive cell source for MPC-based therapeutic applications [25].

Previously, a great deal of attention has been focused on the notion that local delivery of *ex vivo* culture-expanded MPCs will strengthen regeneration of destructive joint tissue associated with OA in clinical trials [26,27]. The efficacy of intra-articular delivery of autologous and allogeneic MPCs in the knee has now been verified in preclinical OA animal models [28,29,30]. Furthermore, xenogeneic cells, human BM-MPCs, had also been demonstrated the effectiveness in a rat and guinea pig spontaneous OA models, respectively [9,31]. However, to our knowledge, the efficacy of xenogeneic adipose-derived MPCs on OA animal model has not been fully investigated.

To date, we are the first to present an investigation delivering xenogeneic human adipose-derived MPCs into the knee joints of rabbit OA model. Xenogeneic haMPCs were chosen because of some considerations: (1) Investigating the effect of haMPCs on osteoarthritis as a cell-therapy candidate in preclinical animal study; (2) The yielding of MPCs in an equal volume of lipoaspirates exceeds bone marrow aspirate by about 300-fold [14,32,33]; (3) In comparison to AD-MPCs, BM-MPCs showed higher expression of angiogenic markers, such as angiopoietin and vascular endothelial growth factor which exhibited inhibitive effect on OA cartilage regeneration [34]; and (4) Telomere length showed no compromise in AD-MPCs during aging [24].

In this study, anterior cruciate ligament transaction (ACLT) combined with complete medial meniscectomy was carried to induce moderate to severe OA conditions to explore the efficacy of haMPCs, although ACLT alone was investigated in other rabbit moderate OA models [35,36,37]. We found that eroded cartilage was almost completely covered by the repaired tissues, and the hyaline cartilage specific marker, collagen II expression was increased in haMPC treatment group compared with HA group. To our surprise, we are the first to find that engraftment of xenogeneic haMPCs into rabbit cartilage. This is consistent with previous investigations showing that engraftment of bone marrow-derived GFP-transduced MPCs were associated with the surface and interior of regenerated caprine meniscal tissue [8] and articular cartilage of guinea pigs [9]. These data highlighted direct engraftment should be one of important mechanisms of actions for intra-articular injections of MPCs for cartilage renovation in OA animal model. 

In the present study, the changes of hyaline and hypertrophic marker, type II collagen, was also investigated after haMPC treatment. Previously, it was found that matrix degradation in early OA may be due to aggrecanases, MMP-3 and ADAMTS-5 (a disintegrin and metalloproteinase with thrombospondin motifs), followed by increased activity of collagenases, MMP-13, which was highly efficient at degrading type II collagen [38]. This was confirmed by the fact that global knockout *Adamts-5^−/−^* mice exhibited protection against OA progression and global knockout *Mmp-13^−/−^* mice showed inhibition of cartilage erosion [22,39]. Recent investigation uncovered that in OA conditions the receptor tyrosine kinase discoidin domain receptor 2 could expose to its ligand, native type II collagen, which in turn induced and activated MMP-13 expression in chondrocytes [40]. Our data demonstrated that haMPCs could prevent type II collagen degraded enzyme MMP-13 secretion and in turn rescue type II collagen expression (Figure 5), consistent with previous investigations [8,31].

To our surprise, after 10 weeks of first dose transplantation, signal of human mitochondrial was detected in superficial zone of rabbit cartilage indicating that human MPCs were engrafted into rabbit cartilage (Figure 6). This was consistent with previous investigations showing that engraftment of GFP-transduced bone marrow-derived mesenchymal stem cells were associated with the surface and interior of regenerated caprine meniscal tissue [8] and articular cartilage of guinea pigs [9]. However, in these aforementioned investigations, BM-MSCs were only detected after five to six weeks of implantation. We are the first to find that engraftment of haMPCs into rabbit cartilage lasted as long as at least 10 weeks after implantation. These data highlighted direct regeneration should be one of important mechanism of actions of intra-articular injection of haMPCs for OA cartilage long-term renovation.

This study still has some limitations. For example, although rabbits were chosen in this study and some other preclinical experiments [37,41,42], rabbits were not upright walking animals and the anatomy of rabbit knee joints were not much close to human beings. In order to solve this problem, sheep models have been used in another experiment investigating the role of MPCs on OA. Another shortcoming is, since the extensive pathologic changes in OA are identified as “joint failure” [43], not only articular cartilage but also changes of synovial membrane, subchondral bone and joint capsule should also be investigated. In our ongoing sheep experiment, inflammatory factors secreted by synovial membrane, such as IL-1, TNF-α, IL-10, and density of subchondral trabecular bone, will be detected. 

## 4. Experimental Section

### 4.1. General Experimental Design

**Figure 8:**
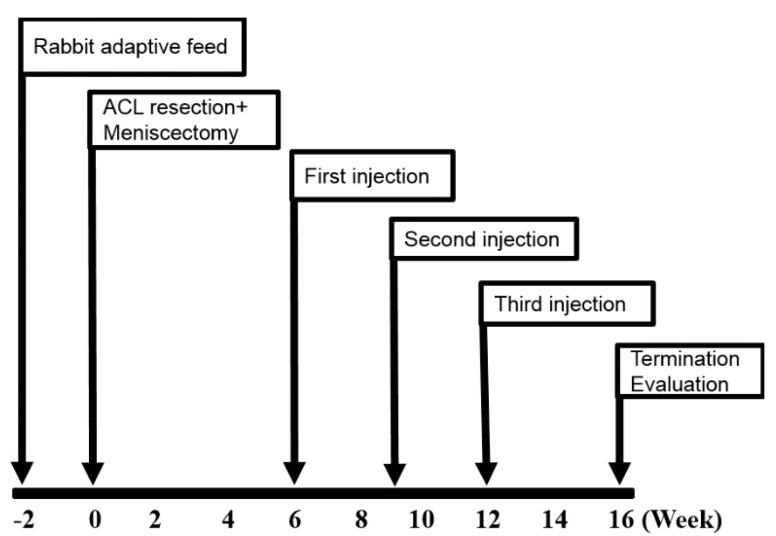
Experimental scheme.

Adult male New Zealand white rabbits (*n* = 12) weighing 2.5 ± 0.5 kg were used. All animals were induced OA by ACLT and medial meniscectomy of right knee. The animals were divided randomly into two groups according to the subsequent intra-articular injections which they received: HA group as vehicle control (*n* = 6) and haMPC group (*n* = 6), and the contralateral knee served as normal group. The overview of the total experimental design was shown in Figure 8. All animal experimental protocol was approved by Ethical Committee of Shanghai Medical College, Fudan University.

### 4.2. OA Model

ACLT and medial meniscectomy of right knee were performed according to previous methods under general anesthesia and sterile conditions [8]. ACL removal was performed by first excising its attachment on the medial aspect of the lateral femoral condyle. The proximal attachment was brought forward and the entire ligament was excised from its tibial attachment. The stifle was moved in a drawer test to ensure that the entire cruciate ligament had been excised. The medial meniscus was removed by sharp excision. The caudal horn of the meniscus was grasped with a hemostat and its axial (lateral) attachment was excised from its tibial attachment. Working from caudal to lateral, then cranial, the meniscus was excised from its attachments until it was completely removed.

### 4.3. Isolation and Culture of haMPCs

CBMG haMPCs were isolated from lipoaspirates. The lipoaspirate (30 g) was washed four times with PBS to remove the red blood cells and tissue debris. The washed tissue was resuspended in 150 mL PBS containing 0.075% *w*/*v* collagenase A type I (Sigma, St. Louis, MO, USA) and incubated on a shaker at 37 °C for 30 min. After centrifugation at 300× *g* for 10 min, the cell pellets were resuspended in the culture medium and passed through a 100 µm filter (BD Biosciences, Mississauga, ON, Canada). Then, the cell pellets were resuspended in serum-free (GibcoBRL, Grand Island, NY, USA) and antibiotic free medium supplemented with 2 mM l-glutamine (GibcoBRL), added to tissue culture flasks, and cultured for 72 h at 37 °C in 5% CO_2_ and 90% humidity. Unattached cells and debris were then removed and fresh medium was added to the adherent cells. The cells were cultured to 80% confluence before being released with trypsin–EDTA and sub-cultured. The medium of the flasks changed every 3 days. Passage five haMPCs were harvested for animal study.

### 4.4. Flow Cytometry Analysis

Cells (1 × 10^6^) were incubated with 1 µg phycoerythrin (PE)-or fluorescein isothiocyanate (FITC)-conjugated monoclonal antibodies specific to human cell surface markers. The antibodies included the following: mouse anti-human CD29, CD34, CD45, CD90, CD14, CD73, CD49d, HLA-DR (BD Pharmingen, San Diego, CA, USA), Anti-alpha smooth muscle Actin [] antibody (FITC) (Abcam, Cambridge, MA, USA), rat anti-human HLA-I (Abcam). A 5-color flow cytometric analysis was performed using an EPICS XL flow cytometer (Beckman Coulter, Palo Alto, CA, USA).

### 4.5. haMPCs Differentiation

The multipotent differentiation potential of haMPCs toward the chondrogenic, osteogenic, and adipogenic lineages was evaluated *in vitro* according to established protocol. Briefly, cells at passage four were seeded onto 12 well plates at 1 × 10^4^ cells per cm^2^ for adipogenesis and 5 × 10^3^ cells per cm^2^ for osteogenesis or at the center of multiwell plate wells with 5 µL droplets of cell solution of 1.6 × 10^7^ for chondrogenesis in DMEM supplemented with penicillin (100 IU/mL)/streptomycin (100 µg/mL) and 10% (vol/vol) FBS (Gibco, Grand Island, NY, USA), incubated at 37 °C in the presence of 5% CO_2_. Two days later, culture medium was replaced with differentiation medium (Gibco, USA), and it was changed every two days thereafter. At 14 days for adipogenesis and 28 days for osteogenesis and chondrogenesis, samples were fixed with 4% paraformaldehyde for 30 min. Positive induction of chondrogenesis, osteogenesis, and adipogenesis was confirmed by Alcian Blue staining, Alizarin Red staining, and Oil Red O staining, respectively.

### 4.6. Intra-Articular Injection of haMPCs

At 6, 9 and 12 weeks after surgery, haMPC group was injected with haMPCs (0.15 mL 2.5 × 10^6^ cells dissolved in 0.15 mL HA), HA group was treated with 0.15 ml cell free medium dissolved in 0.15 mL HA by 25G needles, and normal group received no treatment. At 16 weeks after surgery, all rabbits were sacrificed and femoral condyles were isolated for morphological, histological, and immunohistochemical analyses.

### 4.7. Macroscopic Examination

After rabbits were sacrificed, macroscopic assessment of the knee joint surfaces from the animals was to assess cartilage lesions. The evaluation was analyzed by two blinded investigators, and then scored based on the International Cartilage Research Society (ICRS) for cartilage repair. The ICRS score was designed by Brittberg and Peterson [44,45].

### 4.8. Histological Analysis

After joint surfaces were grossly examined, distal femurs and proximal tibias were excised with the knee joints intact. All samples were fixed in 4% paraformaldehyde and decalcified in EDTA-buffered saline solution (pH 7.4) (0.25 mol/L). After fixation and decalcification, the specimens were cut into four pieces. Specimens were paraffin embedded and thin sections (6 μm) were taken. Hematoxylin/eosin (HE) and Safranin-O/Fast green stainings were used to assess general morphology and proteoglycans/collagen content. All the evaluations were performed by two blinded researchers with an Eclipse 90i microscope (Nikon, Tokyo, Japan), and scored according to Modified O’Driscoll grading system [46].

Moreover, quantitative evaluation of cartilage thickness (CT) was carried out according to previously described methods [47]. CT values stood for the mean of 10 measurements perpendicular to the articular surface measured by micrometer [48].

### 4.9. Immunohistochemistry

All sections were deparaffinized, rehydrated and digested with pepsin for 20 min at 37 °C. The slide was then incubated in hydrogen peroxide block solution for 15 min, and Ultra V Block solution was applied for 5 min at RT. All primary antibodies including rabbit anti-collagen II (1:200; Abcam), rabbit anti-MMP13 (1:200; Abcam), mouse anti-human mitochondria (1:500; Millipore, Billerica, MA, USA), ratanti-human HLA-I (1:200; Abcam) and rabbit anti-human HLA-II-DR (1:200; Abcam), and incubated overnight at 4 °C, respectively. Biotinylated secondary antibody and streptavidin peroxidase solution were applied at RT for 30 and 45 min, separately.

### 4.10. Statistical Analysis

All data are presented as mean ± S.D. Quantitative data were analysed by one-way analysis of variance (ANOVA), followed by Student-Newman-Keuls test and Dunnett’s test. Differences were considered significant when *p* < 0.05.

## 5. Conclusions

In conclusion, intra-articular injections of haMPCs were effective in treating rabbit OA models. Direct engraftment was one of the mechanisms of intra-articular injection of MSCs for OA cartilage renovation. These data demonstrated that MSCs are effective in treating osteoarthritis and supported the fact that MSCs are transplantable between HLA-incompatible individuals.
